# Supplementary material for: Potential Anticancer Activities and Catalytic Oxidation Efficiency of Platinum(IV) Complex
Source: Molecules. 2022 Jul 9;27(14):4406. doi: 10.3390/molecules27144406 (PMC9323151; doi:10.3390/molecules27144406)
Supplement: Supplementary file 1 [file molecules-27-04406-s001.zip › molecules-1796815-supplementary.pdf]

“Supporting Information”

Potential Anticancer Activities and Catalytic Oxidation Efficiency of Platinum(IV) Complex

Mohamed M. El-bendary [1,2,*], Tamer S. Saleh[1,3], Mansour M. Alomari [1], Ehab M. M. Ali [4,5], Bambar Davaasuren [6], Mariusz Jaremko [7], Bandar A. Babgi [8]

1 Department of Chemistry, College of Science, University of Jeddah, Jeddah 21589, Saudi Arabia; tssayed@uj.edu.sa (T.S.S.); 1900066@uj.edu.sa (M.M.A.)

2 Chemistry Department, Faculty of Science, Tanta University, Tanta 31527, Egypt

3 Green Chemistry Department, National research Centre, Dokki, Giza 12622, Egypt

4 Biochemistry Department, Faculty of Science, King Abdulaziz University, Jeddah 21589, Saudi Arabia; emali@kau.edu.sa

5 Division of Biochemistry, Department of Chemistry, Faculty of Science, Tanta University, Tanta 31527, Egypt

6 Core Labs, King Abdullah University of Science and Technology (KAUST), Thuwal 23955-6900, Saudi Arabia; bambar.davaasuren@kaust.edu.sa

7 Biological and Environmental Science and Engineering (BESE) Division, King Abdullah University of Science and Technology (KAUST), Thuwal 23955-6900, Saudi Arabia; mariusz.jaremko@kaust.edu.sa

8 Department of Chemistry, Faculty of Science, King Abdulaziz University, P.O. Box 80203, Jeddah 21589, Saudi Arabia; bbabgi@kau.edu.sa

* Correspondence: mmesmaeel@uj.edu.sa

Keywords: platinum(IV); Pyridine-2-carbaldehyde-oxime; anticancer agent; catalytic oxidation activity; luminescence

2.8. A general procedure for the oxidation of benzyl alcohols:

2.8.1. Sonicated reactions:

A mixture of benzyl alcohol derivatives (5 mmol) and 30 % aqueous hydrogen peroxide (7.5 mmol), the complex **1** catalyst (10 mol%) in a 25ml round bottom flask was sonicated using an Elma sonicator P30H instrument. All the reactions were carried out at 60-70 ^oC, which was maintained by adding or removing the water in an ultrasonic bath (the temperature inside the reaction vessel was 66-68^oC). The sonochemical reactions were continued for a determined time until the starting material was no longer detectable by TLC. Water (5 mL) was added and the mixture was extracted with ethyl acetate (3 × 5 mL). The combined organic layers were dried over sodium sulfate and concentrated under reduced pressure to give pure aldehyde derivatives **2a-c.** The identity of the products was ascertained by comparison with an authentic sample and IR, ^{1}NMR &^{13}C NMR.

2.8.2. Method A: Silent Reaction

The reactions under silent conditions were performed on the same scale and conditions as described for sonicated reactions. The reaction was stirred and carried out at 60-70 ^oC, the reaction was continued for a determined time [until the starting material was no longer detectable by TLC]. After completion of the reaction, the products obtained were purified as described previously in sonicated reaction.

2.8. The synthesized compounds with their physical data

4-Methoxybenzaldehyde (2a)

Colorless liquid; ^{1}H NMR (400 MHz, $CDCl_3$): δ 9.77 (s, 1H), 7.77 (d, *J* = 8.0 Hz, 2H), 7.12 (d, *J* = 8.0 Hz, 2H), 3.83 (s, 3H); ^{13}CNMR (100 MHz, $CDCl_3$): δ 190.11, 164.5, 131.9, 129.8, 114.3, 55.4.

4-Chlorobenzaldehyde (2b)

White solid; ^{1}HNMR (400 MHz, $CDCl_3$): δ 9.79 (s, 1H), 8.00 (d, *J* = 8.0Hz, 2H), 7.84 (d, *J* = 8.0Hz, 2H); ^{13}CNMR (100MHz, $CDCl_3$): δ 190.9, 129.5, 128.9, 130.9, 131.5.

4-Bromobenzaldehyde (2c)

Off white crystals; ^{1}HNMR (400 MHz, $CDCl_3$): δ 9.91 (s, 1H), 8.02 (d, *J* = 8.0Hz, 2H), 7.81 (d, *J* = 8.0Hz, 2H); ^{13}CNMR (100MHz, $CDCl_3$): δ 190.9, 129.8, 129.4, 131.9, 134.5.

Table S1 Crystal data and structure refinement for complex 1

Empirical formula	$C_{14}H_{10}N_6O_2PtS_2$
CCDC	2096540
Formula weight	553.49
Temperature/K	120
Crystal system	monoclinic
Space group	$P2_1/n$
a, b, c /Å	6.4862(2), 7.8537(2), 15.9544(5)
β/°	99.0950(10)
Volume/Å^3	802.51(4)
Z	2
ρ_{calc}g/cm^3	2.291
μ/mm^{-1}	9.025
F(000)	524.0
Crystal size/mm^3	0.217 × 0.064 × 0.06
Radiation	MoKα (λ = 0.71073)
2Θ range for data collection/°	7.326 to 120.324
Index ranges	-15 ≤ h ≤ 15, -19 ≤ k ≤ 19, -38 ≤ l ≤ 38
Reflections collected	207792
Independent reflections	12182 [R_{int} = 0.0341, R_{sigma} = 0.0125]
Data/restraints/parameters	12182/0/115
Goodness-of-fit on F^2	1.110
Final R indexes [I>=2σ (I)]	R_1 = 0.0153, wR_2 = 0.0332
Final R indexes [all data]	R_1 = 0.0220, wR_2 = 0.0360
Largest diff. peak/hole / e Å^{-3}	2.88/-1.42

Table S2. Selected bond lengths and angles for the platinum complex 1

Atom	Atom	Length/Å	Atom	Atom	Atom	Angles/ ^{0}
S1	C1	1.6745(7)	S1	Pt1	S1[1]	180.0
S1	Pt1	2.38012(19)	N2	Pt1	S1	87.589(17)
O1	N2	1.2678(8)	N2[1]	Pt1	S1[1]	87.589(17)
N1	C1	1.1613(10)	N2	Pt1	S1[1]	92.410(17)
N2	C7	1.3124(9)	N2[1]	Pt1	S1	92.411(17)
N2	Pt1	2.0767(5)	N2[1]	Pt1	N2	180.00(2)
N3	C2	1.3406(8)	N3[1]	Pt1	S1	87.597(17)
N3	C6	1.3619(8)	N3[1]	Pt1	S1[1]	92.403(17)
N3	Pt1	2.0414(5)	N3	Pt1	S1[1]	87.596(17)
			N3	Pt1	S1	92.404(17)
			N3[1]	Pt1	N2	100.76(2)
			N3	Pt1	N2	79.24(2)
			N3[1]	Pt1	N2[1]	79.24(2)
			N3	Pt1	N2[1]	100.76(2)
[1] = 1-X,1-Y,1-Z			N3[1]	Pt1	N3	180.00(3)

Table S3. Hydrogen bond lengths (Å) and bond angles (deg.) in complex **1**

**D-H…A**	**d(D-H)**	**d(H…A)**	**d(D…A)**	**∧(DHA)**
C3–H3…N1	0.950	2.441	3.385	172.39
C7–H7…N1	0.950	2.721	3.499	139.62
C5 –H5…Ol	0.950	2.362	3.141	138.85
C5 –H5…N1	0.950	2.866	3.639	139.26
C4 –H4…O1	0.950	3.068	3.483	108.51

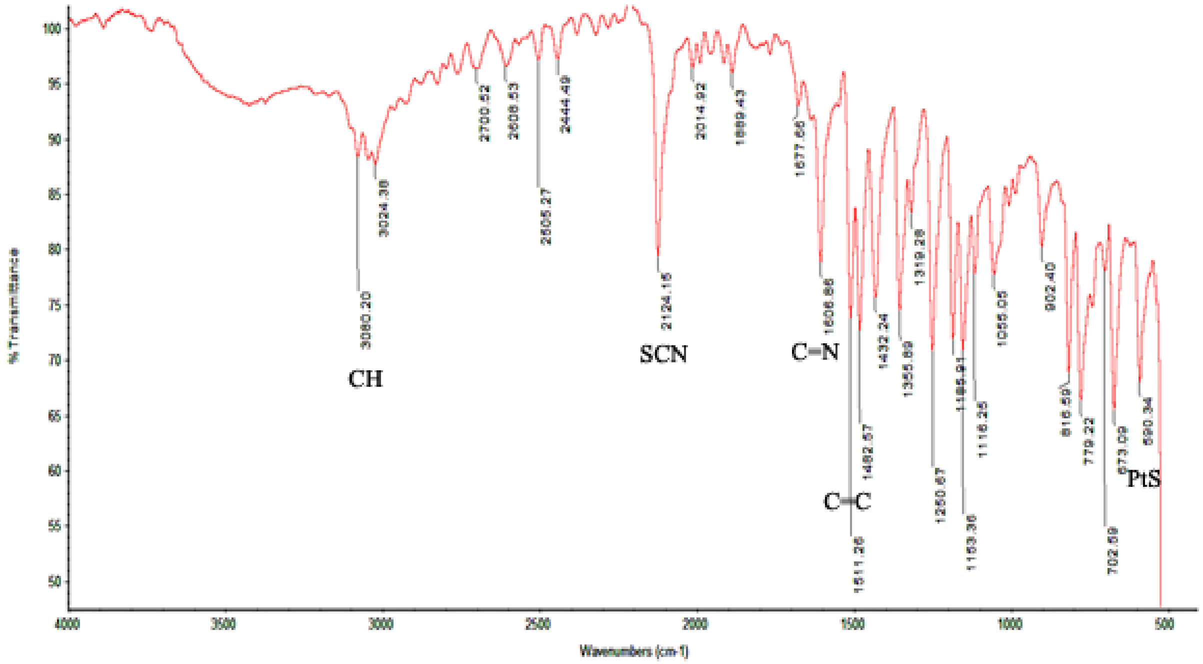

Fig.S1. IR spectrum of complex **1**

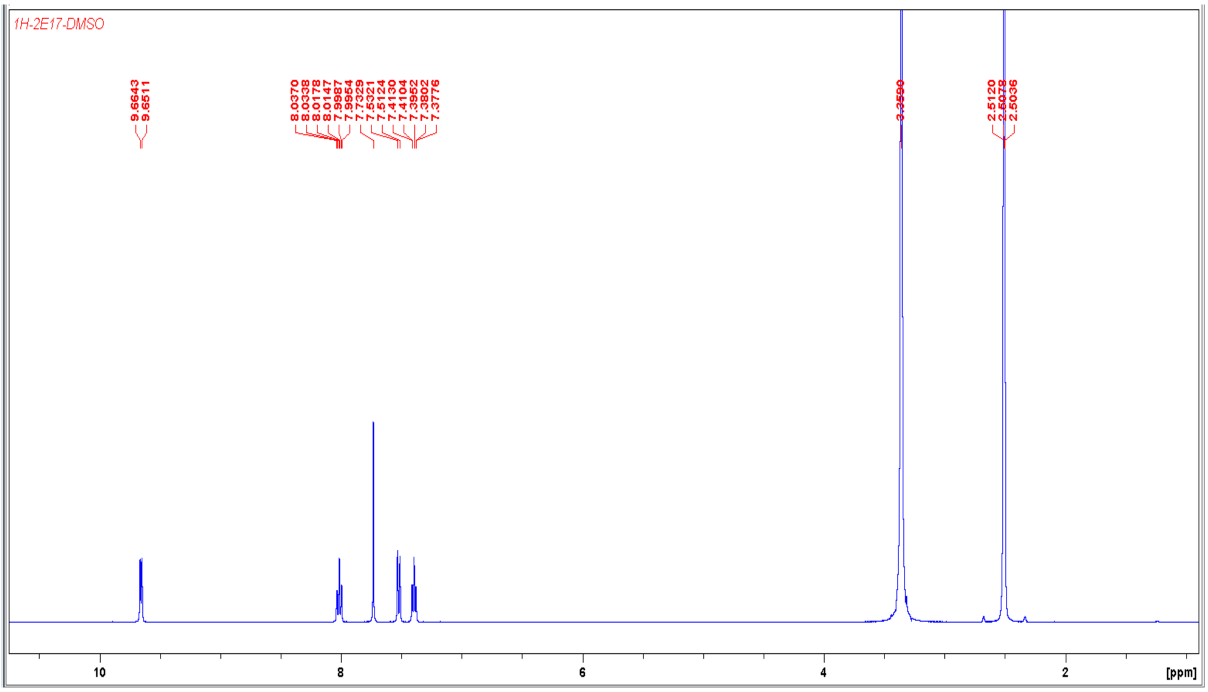

Figure S2. ^{1}H-NMR spectrum of complex **1**

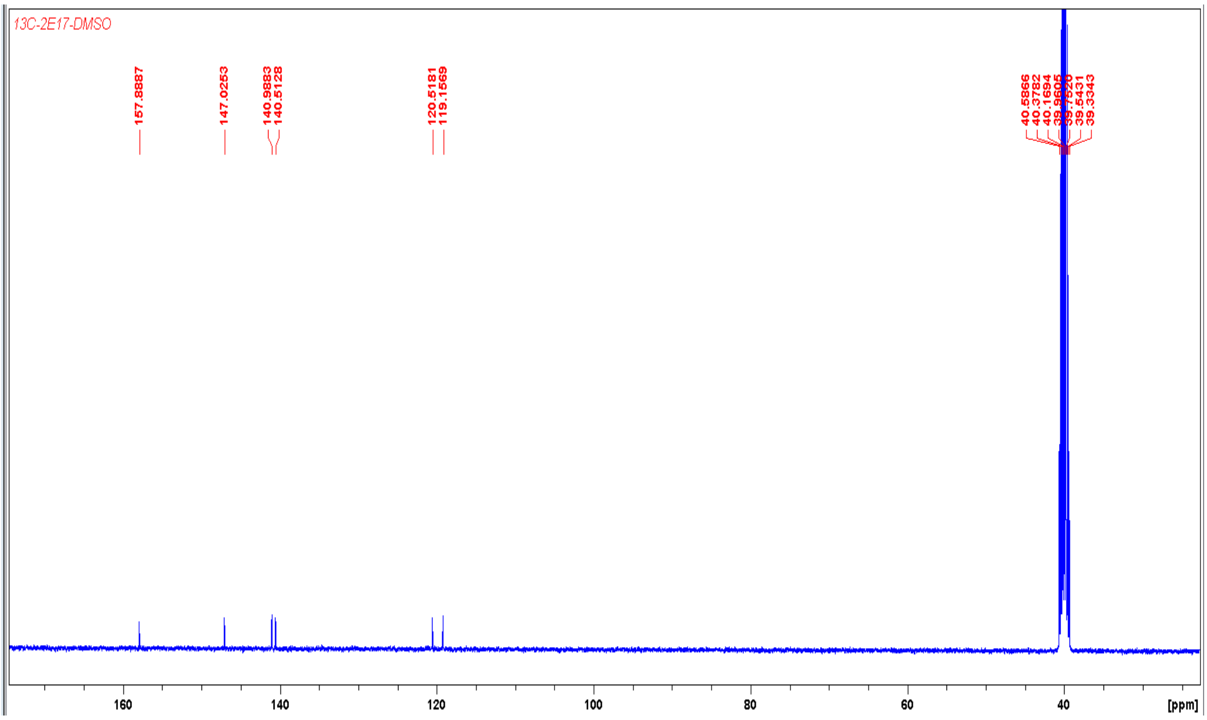

Figure S3. ^{13}C-NMR spectrum of complex **1**

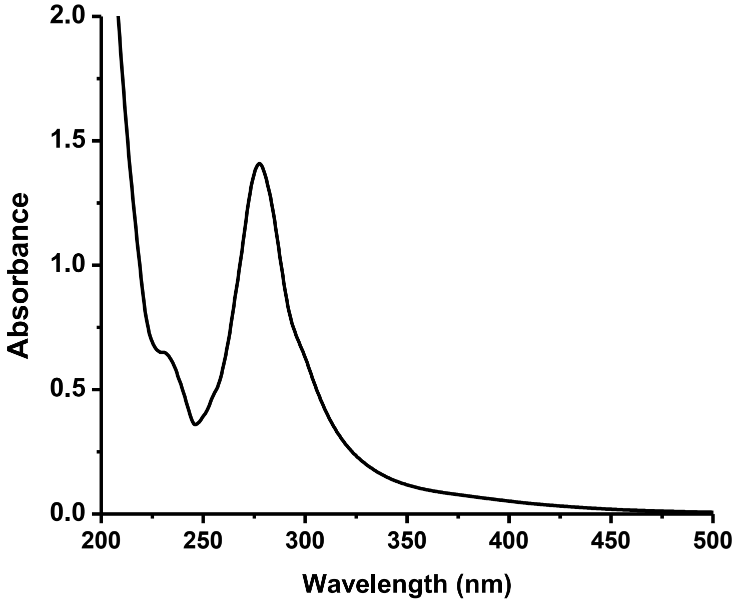

Figure S4. UV absorption spectrum of the complex **1**

Full cif file for complex 1:

data_a3_2_mon_a

_audit_creation_date 2021-07-14

_audit_creation_method;

Olex2 1.3

(compiled 2020.11.12 svn.r5f609507 for OlexSys, GUI svn.r6272);

_shelx_SHELXL_version_number '2018/3'

loop_

_audit_author_name

_audit_author_email

_audit_author_address

'Davaasuren, B.' bambar.davaasuren@kaust.edu.sa

;

King Abdullah University of Science and Technology

Core Labs

KAUST

Thuwal

23955-6900

Kingdom of Saudi Arabia;

_audit_contact_author_address ?

_audit_contact_author_email ?

_audit_contact_author_name ''

_audit_contact_author_phone ?

_publ_contact_author_id_orcid ?

_publ_section_references

;

Dolomanov, O.V., Bourhis, L.J., Gildea, R.J, Howard, J.A.K. & Puschmann, H.

(2009), J. Appl. Cryst. 42, 339-341.

Sheldrick, G.M. (2015). Acta Cryst. A71, 3-8.

Sheldrick, G.M. (2015). Acta Cryst. C71, 3-8.;

_chemical_name_common ?

_chemical_name_systematic ?

_chemical_formula_moiety 'C14 H10 N6 O2 Pt S2'

_chemical_formula_sum 'C14 H10 N6 O2 Pt S2'

_chemical_formula_weight 553.49

_chemical_melting_point ?

loop_

_atom_type_symbol

_atom_type_description

_atom_type_scat_dispersion_real

_atom_type_scat_dispersion_imag

_atom_type_scat_source

'C' 'C' 0.0033 0.0016 'International Tables Vol C Tables 4.2.6.8 and 6.1.1.4'

'H' 'H' 0.0000 0.0000 'International Tables Vol C Tables 4.2.6.8 and 6.1.1.4'

'N' 'N' 0.0061 0.0033 'International Tables Vol C Tables 4.2.6.8 and 6.1.1.4'

'O' 'O' 0.0106 0.0060 'International Tables Vol C Tables 4.2.6.8 and 6.1.1.4'

'Pt' 'Pt' -1.7033 8.3905

'International Tables Vol C Tables 4.2.6.8 and 6.1.1.4'

'S' 'S' 0.1246 0.1234 'International Tables Vol C Tables 4.2.6.8 and 6.1.1.4'

_shelx_space_group_comment;

The symmetry employed for this shelxl refinement is uniquely defined

by the following loop, which should always be used as a source of

symmetry information in preference to the above space-group names.

They are only intended as comments.;

_space_group_crystal_system 'monoclinic'

_space_group_IT_number 14

_space_group_name_H-M_alt 'P 1 21/n 1'

_space_group_name_Hall '-P 2yn'

loop_

_space_group_symop_operation_xyz

'x, y, z'

'-x+1/2, y+1/2, -z+1/2'

'-x, -y, -z'

'x-1/2, -y-1/2, z-1/2'

_cell_length_a 6.4862(2)

_cell_length_b 7.8537(2)

_cell_length_c 15.9544(5)

_cell_angle_alpha 90

_cell_angle_beta 99.0950(10)

_cell_angle_gamma 90

_cell_volume 802.51(4)

_cell_formula_units_Z 2

_cell_measurement_reflns_used 9761

_cell_measurement_temperature 120.02

_cell_measurement_theta_max 60.06

_cell_measurement_theta_min 3.66

_shelx_estimated_absorpt_T_max 0.613

_shelx_estimated_absorpt_T_min 0.245

_exptl_absorpt_coefficient_mu 9.025

_exptl_absorpt_correction_T_max 0.7379

_exptl_absorpt_correction_T_min 0.3505

_exptl_absorpt_correction_type numerical

_exptl_absorpt_process_details

;

SADABS-2016/2 (Bruker,2016/2) was used for absorption correction.

wR2(int) was 0.0901 before and 0.0476 after correction.

The Ratio of minimum to maximum transmission is 0.4750.

The \l/2 correction factor is Not present.

;

_exptl_absorpt_special_details ?

_exptl_crystal_colour 'clear orangish brown'

_exptl_crystal_colour_lustre clear

_exptl_crystal_colour_modifier orangish

_exptl_crystal_colour_primary brown

_exptl_crystal_density_diffrn 2.291

_exptl_crystal_density_meas ?

_exptl_crystal_density_method ?

_exptl_crystal_description block

_exptl_crystal_F_000 524

_exptl_crystal_size_max 0.217

_exptl_crystal_size_mid 0.064

_exptl_crystal_size_min 0.06

_exptl_transmission_factor_max ?

_exptl_transmission_factor_min ?

_diffrn_reflns_av_R_equivalents 0.0341

_diffrn_reflns_av_unetI/netI 0.0125

_diffrn_reflns_Laue_measured_fraction_full 0.981

_diffrn_reflns_Laue_measured_fraction_max 0.998

_diffrn_reflns_limit_h_max 15

_diffrn_reflns_limit_h_min -15

_diffrn_reflns_limit_k_max 19

_diffrn_reflns_limit_k_min -19

_diffrn_reflns_limit_l_max 38

_diffrn_reflns_limit_l_min -38

_diffrn_reflns_number 207792

_diffrn_reflns_point_group_measured_fraction_full 0.981

_diffrn_reflns_point_group_measured_fraction_max 0.998

_diffrn_reflns_theta_full 25.242

_diffrn_reflns_theta_max 60.162

_diffrn_reflns_theta_min 3.663

_diffrn_ambient_temperature 120.02

_diffrn_detector 'Bruker APEX2 area detector'

_diffrn_detector_area_resol_mean 7.9

_diffrn_detector_type 'CCD area detector'

_diffrn_measured_fraction_theta_full 0.981

_diffrn_measured_fraction_theta_max 0.998

_diffrn_measurement_device 'three-circle diffractometer'

_diffrn_measurement_device_type 'Bruker SMART APEX2 area detector'

_diffrn_measurement_method '\w and \f scans'

_diffrn_radiation_monochromator 'mirror optics'

_diffrn_radiation_probe x-ray

_diffrn_radiation_type MoK\a

_diffrn_radiation_wavelength 0.71073

_diffrn_source 'microfocus sealed X-ray tube'

_diffrn_source_current 1.0

_diffrn_source_power 0.05

_diffrn_source_type 'Incoatec I\ms'

_diffrn_source_voltage 50.0

_diffrn_standards_number 0

_reflns_Friedel_coverage 0.000

_reflns_Friedel_fraction_full .

_reflns_Friedel_fraction_max .

_reflns_number_gt 10035

_reflns_number_total 12182

_reflns_special_details
;
 Reflections were merged by SHELXL according to the crystal
 class for the calculation of statistics and refinement.

 _reflns_Friedel_fraction is defined as the number of unique
 Friedel pairs measured divided by the number that would be
 possible theoretically, ignoring centric projections and
 systematic absences.
;
_reflns_threshold_expression 'I > 2\s(I)'
_computing_cell_refinement ?
_computing_data_collection ?
_computing_data_reduction ?
_computing_molecular_graphics 'Olex2 1.3 (Dolomanov et al., 2009)'
_computing_publication_material 'Olex2 1.3 (Dolomanov et al., 2009)'
_computing_structure_refinement 'SHELXL 2018/3 (Sheldrick, 2015)'
_computing_structure_solution 'SHELXT 2018/2 (Sheldrick, 2018)'
_refine_diff_density_max 2.875
_refine_diff_density_min -1.419
_refine_diff_density_rms 0.140
_refine_ls_extinction_coef .
_refine_ls_extinction_method none
_refine_ls_goodness_of_fit_ref 1.110
_refine_ls_hydrogen_treatment constr
_refine_ls_matrix_type full
_refine_ls_number_parameters 115
_refine_ls_number_reflns 12182
_refine_ls_number_restraints 0
_refine_ls_R_factor_all 0.0220
_refine_ls_R_factor_gt 0.0153
_refine_ls_restrained_S_all 1.110
_refine_ls_shift/su_max 0.002
_refine_ls_shift/su_mean 0.000
_refine_ls_structure_factor_coef Fsqd
_refine_ls_weighting_details
 'w=1/[\s^2^(Fo^2^)+(0.0127P)^2^+0.3756P] where P=(Fo^2^+2Fc^2^)/3'

_refine_ls_weighting_scheme calc
_refine_ls_wR_factor_gt 0.0332
_refine_ls_wR_factor_ref 0.0360
_refine_special_details ?
_olex2_refinement_description
;
1. Fixed Uiso
 At 1.2 times of:
  All C(H) groups
2.a Aromatic/amide H refined with riding coordinates:
 C2(H2), C3(H3), C4(H4), C5(H5), C7(H7)
;
_atom_sites_solution_hydrogens geom
_atom_sites_solution_primary dual
_atom_sites_solution_secondary ?
loop_
  _atom_site_label
  _atom_site_type_symbol
  _atom_site_fract_x
  _atom_site_fract_y
  _atom_site_fract_z
  _atom_site_U_iso_or_equiv
  _atom_site_adp_type
  _atom_site_occupancy
  _atom_site_site_symmetry_order
  _atom_site_calc_flag
  _atom_site_refinement_flags_posn
  _atom_site_refinement_flags_adp
  _atom_site_refinement_flags_occupancy
  _atom_site_disorder_assembly
  _atom_site_disorder_group
 S1 S 0.66549(3) 0.72868(2) 0.43873(2) 0.01198(2) Uani 1 1 d . . . . .
 O1 O 0.92334(8) 0.43191(8) 0.59502(4) 0.01412(8) Uani 1 1 d . . . . .
 N1 N 0.32322(14) 0.94997(11) 0.38793(6) 0.02208(14) Uani 1 1 d . . . . .
 N2 N 0.74948(9) 0.50254(7) 0.59917(3) 0.01031(6) Uani 1 1 d . . . . .
 N3 N 0.38692(9) 0.65795(7) 0.58365(3) 0.00999(6) Uani 1 1 d . . . . .
 C1 C 0.46162(11) 0.85856(9) 0.41050(5) 0.01344(9) Uani 1 1 d . . . . .

C2 C 0.20422(11) 0.74109(9) 0.56938(5) 0.01327(9) Uani 1 1 d . . . . .

H2 H 0.119237 0.732873 0.515287 0.016 Uiso 1 1 calc R U . . .

C3 C 0.13706(13) 0.83910(11) 0.63240(6) 0.01670(11) Uani 1 1 d . . . . .

H3 H 0.009508 0.900553 0.621254 0.020 Uiso 1 1 calc R U . . .

C4 C 0.26025(15) 0.84557(11) 0.71223(5) 0.01745(12) Uani 1 1 d . . . . .

H4 H 0.214880 0.908895 0.756664 0.021 Uiso 1 1 calc R U . . .

C5 C 0.44889(13) 0.75956(10) 0.72671(5) 0.01495(10) Uani 1 1 d . . . . .

H5 H 0.533591 0.762719 0.781007 0.018 Uiso 1 1 calc R U . . .

C6 C 0.51253(11) 0.66835(8) 0.66041(4) 0.01100(8) Uani 1 1 d . . . . .

C7 C 0.71058(11) 0.58405(9) 0.66676(4) 0.01242(8) Uani 1 1 d . . . . .

H7 H 0.809108 0.587083 0.717578 0.015 Uiso 1 1 calc R U . . .

Pt1 Pt 0.500000 0.500000 0.500000 0.00715(1) Uani 1 2 d S . P A 1

loop_

_atom_site_aniso_label

_atom_site_aniso_U_11

_atom_site_aniso_U_22

_atom_site_aniso_U_33

_atom_site_aniso_U_23

_atom_site_aniso_U_13

_atom_site_aniso_U_12

S1 0.00979(5) 0.01231(5) 0.01423(6) 0.00274(5) 0.00311(4) 0.00171(4)

O1 0.00946(15) 0.0193(2) 0.01315(17) 0.00093(15) 0.00040(12) 0.00499(14)

N1 0.0181(3) 0.0185(3) 0.0293(4) 0.0071(3) 0.0024(3) 0.0068(2)

N2 0.00977(14) 0.01236(16) 0.00846(13) 0.00071(14) 0.00037(11) 0.00224(13)

N3 0.01042(15) 0.01086(16) 0.00890(15) -0.00082(12) 0.00216(12) 0.00259(12)

C1 0.0131(2) 0.0125(2) 0.0148(2) 0.00282(17) 0.00226(17) 0.00233(17)

C2 0.0117(2) 0.0144(2) 0.0142(2) -0.00081(18) 0.00343(16) 0.00416(17)

C3 0.0166(3) 0.0161(3) 0.0189(3) -0.0026(2) 0.0073(2) 0.0053(2)

C4 0.0224(3) 0.0155(3) 0.0163(3) -0.0036(2) 0.0090(2) 0.0030(2)

C5 0.0205(3) 0.0143(2) 0.0107(2) -0.00332(17) 0.00426(19) 0.0010(2)

C6 0.0131(2) 0.01140(19) 0.00859(17) -0.00133(14) 0.00184(14) 0.00097(15)

C7 0.0124(2) 0.0154(2) 0.00890(18) -0.00124(16) -0.00029(15) 0.00174(17)

Pt1 0.00718(1) 0.00836(1) 0.00586(1) 0.00012(1) 0.00083(1) 0.00217(1)

_geom_special_details;

All esds (except the esd in the dihedral angle between two l.s. planes)

are estimated using the full covariance matrix. The cell esds are taken

into account individually in the estimation of esds in distances, angles
and torsion angles; correlations between esds in cell parameters are only
used when they are defined by crystal symmetry. An approximate (isotropic)
treatment of cell esds is used for estimating esds involving l.s. planes.;

```
loop_
 _geom_bond_atom_site_label_1
 _geom_bond_atom_site_label_2
 _geom_bond_distance
 _geom_bond_site_symmetry_2
 _geom_bond_publ_flag
S1 C1 1.6745(7) . ?
S1 Pt1 2.38012(19) . ?
O1 N2 1.2678(8) . ?
N1 C1 1.1613(10) . ?
N2 C7 1.3124(9) . ?
N2 Pt1 2.0767(5) . ?
N3 C2 1.3406(8) . ?
N3 C6 1.3619(8) . ?
N3 Pt1 2.0414(5) . ?
C2 H2 0.9500 . ?
C2 C3 1.3899(10) . ?
C3 H3 0.9500 . ?
C3 C4 1.3933(13) . ?
C4 H4 0.9500 . ?
C4 C5 1.3847(12) . ?
C5 H5 0.9500 . ?
C5 C6 1.3929(9) . ?
C6 C7 1.4345(10) . ?
C7 H7 0.9500 . ?

loop_
 _geom_angle_atom_site_label_1
 _geom_angle_atom_site_label_2
 _geom_angle_atom_site_label_3
 _geom_angle
 _geom_angle_site_symmetry_1
 _geom_angle_site_symmetry_3
```

_geom_angle_publ_flag

C1 S1 Pt1 100.48(3) . . ?

O1 N2 C7 122.98(6) . . ?

O1 N2 Pt1 123.26(4) . . ?

C7 N2 Pt1 113.76(4) . . ?

C2 N3 C6 120.70(6) . . ?

C2 N3 Pt1 125.75(5) . . ?

C6 N3 Pt1 113.50(4) . . ?

N1 C1 S1 177.27(8) . . ?

N3 C2 H2 119.4 . . ?

N3 C2 C3 121.11(7) . . ?

C3 C2 H2 119.4 . . ?

C2 C3 H3 120.6 . . ?

C2 C3 C4 118.78(7) . . ?

C4 C3 H3 120.6 . . ?

C3 C4 H4 120.0 . . ?

C5 C4 C3 119.91(7) . . ?

C5 C4 H4 120.0 . . ?

C4 C5 H5 120.5 . . ?

C4 C5 C6 118.97(7) . . ?

C6 C5 H5 120.5 . . ?

N3 C6 C5 120.45(6) . . ?

N3 C6 C7 115.95(5) . . ?

C5 C6 C7 123.60(6) . . ?

N2 C7 C6 116.96(6) . . ?

N2 C7 H7 121.5 . . ?

C6 C7 H7 121.5 . . ?

S1 Pt1 S1 180.0 . 3_666 ?

N2 Pt1 S1 87.589(17) 3_666 3_666 ?

N2 Pt1 S1 92.410(17) . 3_666 ?

N2 Pt1 S1 87.589(17) . . ?

N2 Pt1 S1 92.411(17) 3_666 . ?

N2 Pt1 N2 180.00(2) 3_666 . ?

N3 Pt1 S1 92.403(17) 3_666 3_666 ?

N3 Pt1 S1 87.596(17) . 3_666 ?

N3 Pt1 S1 87.597(17) 3_666 . ?

N3 Pt1 S1 92.404(17) . . ?

N3 Pt1 N2 100.76(2) 3_666 . ?

N3 Pt1 N2 100.76(2) . 3_666 ?

N3 Pt1 N2 79.24(2) . . ?

N3 Pt1 N2 79.24(2) 3_666 3_666 ?

N3 Pt1 N3 180.00(3) 3_666 . ?

loop_

_geom_hbond_atom_site_label_D

_geom_hbond_atom_site_label_H

_geom_hbond_atom_site_label_A

_geom_hbond_distance_DH

_geom_hbond_distance_HA

_geom_hbond_distance_DA

_geom_hbond_angle_DHA

_geom_hbond_site_symmetry_A

_geom_hbond_publ_flag

C2 H2 O1 0.95 2.17 2.9537(10) 139.4 3_666 yes

C3 H3 N1 0.95 2.44 3.3847(11) 172.4 3_576 yes

C5 H5 O1 0.95 2.36 3.1406(9) 138.8 2_656 yes

loop_

_geom_torsion_atom_site_label_1

_geom_torsion_atom_site_label_2

_geom_torsion_atom_site_label_3

_geom_torsion_atom_site_label_4

_geom_torsion

_geom_torsion_site_symmetry_1

_geom_torsion_site_symmetry_2

_geom_torsion_site_symmetry_3

_geom_torsion_site_symmetry_4

_geom_torsion_publ_flag

O1 N2 C7 C6 175.59(7) . . . . ?

N3 C2 C3 C4 -2.01(13) . . . . ?

N3 C6 C7 N2 -1.46(10) . . . . ?

C2 N3 C6 C5 2.88(10) . . . . ?

C2 N3 C6 C7 -175.82(7) . . . . ?

C2 C3 C4 C5 2.01(13) . . . . ?

C3 C4 C5 C6 0.34(13) . . . . ?

C4 C5 C6 N3 -2.81(11) . . . . ?

C4 C5 C6 C7 175.79(8) . . . . ?

C5 C6 C7 N2 179.89(7) . . . . ?

C6 N3 C2 C3 -0.43(11) . . . . ?

Pt1 N2 C7 C6 -4.47(8) . . . . ?

Pt1 N3 C2 C3 176.72(6) . . . . ?

Pt1 N3 C6 C5 -174.60(6) . . . . ?

Pt1 N3 C6 C7 6.70(8) . . . . ?

_shelx_res_file;

TITL A3_2_mon_a.res in P2(1)/n

a3_2_mon_a.res

created by SHELXL-2018/3 at 12:20:02 on 14-Jul-2021

REM Old TITL A3_2_mon in P2(1)/n

REM SHELXT solution in P2(1)/n: R1 0.041, Rweak 0.001, Alpha 0.019

REM <I/s> 2.198 for 128 systematic absences, Orientation as input

REM Formula found by SHELXT: C16 O6 S2 Pt

CELL 0.71073 6.4862 7.8537 15.9544 90 99.095 90

ZERR 2 0.0002 0.0002 0.0005 0 0.001 0

LATT 1

SYMM 0.5-X,0.5+Y,0.5-Z

SFAC C H N O Pt S

UNIT 28 20 12 4 2 4

EQIV $1 1-X,1-Y,1-Z

EQIV $2 -X,2-Y,1-Z

EQIV $3 1.5-X,0.5+Y,1.5-Z

L.S. 40

PLAN 10

SIZE 0.06 0.064 0.217

TEMP -153.13

CONF

HTAB C2 O1_$1

HTAB C3 N1_$2

HTAB C5 O1_$3

HTAB

BOND $H

LIST 4

```
MORE -1
fmap 2
acta
REM <olex2.extras>
REM <HklSrc "%.\\A3_2_mon_a.hkl">
REM </olex2.extras>

WGHT    0.012700    0.375600
FVAR       0.58255
S1    6    0.665495    0.728676    0.438731    11.00000    0.00979    0.01231 =
         0.01423    0.00274    0.00311    0.00171
O1    4    0.923339    0.431911    0.595022    11.00000    0.00946    0.01930 =
         0.01315    0.00093    0.00040    0.00499
N1    3    0.323218    0.949970    0.387927    11.00000    0.01809    0.01847 =
         0.02925    0.00713    0.00241    0.00680
N2    3    0.749480    0.502543    0.599165    11.00000    0.00977    0.01236 =
         0.00846    0.00071    0.00037    0.00224
N3    3    0.386925    0.657952    0.583651    11.00000    0.01042    0.01086 =
         0.00890   -0.00082    0.00216    0.00259
C1    1    0.461618    0.858564    0.410504    11.00000    0.01309    0.01246 =
         0.01480    0.00282    0.00226    0.00233
C2    1    0.204225    0.741089    0.569382    11.00000    0.01169    0.01438 =
         0.01420   -0.00081    0.00343    0.00416
AFIX  43
H2    2    0.119237    0.732873    0.515287    11.00000   -1.20000
AFIX   0
C3    1    0.137059    0.839101    0.632401    11.00000    0.01660    0.01610 =
         0.01886   -0.00263    0.00730    0.00525
AFIX  43
H3    2    0.009508    0.900553    0.621254    11.00000   -1.20000
AFIX   0
C4    1    0.260248    0.845567    0.712229    11.00000    0.02242    0.01553 =
         0.01633   -0.00364    0.00903    0.00303
AFIX  43
H4    2    0.214880    0.908895    0.756664    11.00000   -1.20000
AFIX   0
C5    1    0.448889    0.759563    0.726707    11.00000    0.02049    0.01430 =
```

```
        0.01065   -0.00332    0.00426    0.00105
AFIX  43
H5    2    0.533591    0.762719    0.781007    11.00000   -1.20000
AFIX   0
C6    1    0.512526    0.668349    0.660415    11.00000    0.01306    0.01140 =
        0.00859   -0.00133    0.00184    0.00097
C7    1    0.710583    0.584050    0.666761    11.00000    0.01238    0.01535 =
        0.00890   -0.00124   -0.00029    0.00174
AFIX  43
H7    2    0.809108    0.587083    0.717578    11.00000   -1.20000
AFIX   0
PART 1
PT1   5    0.500000    0.500000    0.500000    10.50000    0.00718    0.00836 =
        0.00586    0.00012    0.00083    0.00217
HKLF 4
REM  A3_2_mon_a.res in P2(1)/n
REM wR2 = 0.0360, GooF = S = 1.110, Restrained GooF = 1.110 for all data
REM R1 = 0.0153 for 10035 Fo > 4sig(Fo) and 0.0220 for all 12182 data
REM 115 parameters refined using 0 restraints
END
WGHT      0.0127      0.3757
REM Instructions for potential hydrogen bonds
EQIV $4 x-1, y, z
HTAB C2 S1_$4
HTAB C2 O1_$1
HTAB C3 N1_$2
HTAB C5 O1_$3
REM Highest difference peak  2.875,  deepest hole -1.419,  1-sigma level  0.140
Q1    1   0.4557  0.4615  0.4841  11.00000  0.05    2.87
Q2    1   0.5509  0.5073  0.4838  11.00000  0.05    2.45
Q3    1   0.4479  0.5186  0.4842  11.00000  0.05    2.36
Q4    1   0.4834  0.5189  0.4602  11.00000  0.05    1.36
Q5    1   0.4789  0.5733  0.5031  11.00000  0.05    1.35
Q6    1   0.4114  0.9206  0.4000  11.00000  0.05    0.86
Q7    1   0.6315  0.6174  0.6650  11.00000  0.05    0.75
Q8    1   0.1608  0.7602  0.5923  11.00000  0.05    0.61
Q9    1   0.6217  0.7244  0.4142  11.00000  0.05    0.58
```

Q10 1 0.4659 0.6851 0.6869 11.00000 0.05 0.58;

.
